# Longitudinal Immunological Analysis of Portuguese Healthcare Workers Across the COVID-19 Pandemic Reveals Differences in the Humoral Immune Response to Vaccines

**DOI:** 10.3390/vaccines12121358

**Published:** 2024-11-30

**Authors:** Luísa Vilela, Anabela Silva, Alberta Cruz, Madalena Sousa, Margarida Costa, Fernando Fonseca, Susana Campino, Taane G. Clark, Anabela Miranda

**Affiliations:** 1 Local Health Unit Póvoa de Varzim/Vila do Conde, Largo da Misericórdia, 4490-421 Póvoa de Varzim, Portugal; 2 Faculty of Infectious and Tropical Diseases, London School of Hygiene & Tropical Medicine, Keppel Street, London WC1E 7HT, UK; susana.campino@lshtm.ac.uk (S.C.); taane.clark@lshtm.ac.uk (T.G.C.); 3 Faculty of Epidemiology and Population Health, London School of Hygiene & Tropical Medicine, Keppel Street, London WC1E 7HT, UK; 4 Department of Infectious Diseases, National Institute of Health Doctor Ricardo Jorge, Public Health Centre Doutor Gonçalves Ferreira, Rua Alexandre Herculano 321, 4000-055 Porto, Portugal

**Keywords:** COVID-19, SARS-CoV-2, vaccines, booster, immunogenicity, antibody titre, prospective cohort study

## Abstract

**Background:** A vaccination programme against severe acute respiratory syndrome coronavirus 2 was initiated in Portugal in December 2020. In this study, we report the findings of a prospective cohort study implemented with the objective of monitoring antibody production in response to COVID-19 vaccination. **Methods:** The humoral immune response to vaccination was followed up using blood samples collected from 191 healthcare workers. Participants were split into three groups: the Oxford-AstraZeneca (Vaxzevria) vaccine group (*n* = 68), the Pfizer-BioNTech COVID-19 (Comirnaty) vaccine group (*n* = 51), and the Post-COVID group (*n* = 72). The kinetics of anti-spike antibody production were evaluated until 56 days on average after the third dose (booster). **Results:** We observed that antibody titres peaked approximately one month after full vaccination and declined steadily thereafter. We also found that mRNA vaccination induces higher titres of antibodies than viral vector vaccination, and both generate greater antibody responses than mild or moderate COVID-19. Additionally, whilst the booster for the Oxford-AstraZeneca and Pfizer-BioNTech groups led to antibody levels higher than those at any previous sample collection point, the booster for the Post-COVID group (persons with a history of COVID-19 prior to vaccination) led to antibody levels lower than those attained one month after the second dose. **Interpretation:** Our results indicate that there are different kinetics of antibody production between individuals who received the Pfizer-BioNtech mRNA vaccine and those who received the Oxford-AstraZeneca vector vaccine, or individuals who had COVID-19 before being vaccinated. Additionally, we observed that exposure to either natural infection or vaccination modulates the response to subsequent vaccination. This is particularly evident after administration of the third dose to the Post-COVID group, where our findings point to a hindrance in vaccine boosting, probably due to unwanted feedback by high titres of pre-existing antibodies.

## 1. Introduction

Severe acute respiratory syndrome coronavirus 2 (SARS-CoV-2), a novel RNA coronavirus, was identified in early January 2020 as the cause of a pneumonia epidemic affecting the city of Wuhan, from where it spread across China, then to European countries, the USA, and the rest of the world. The World Health Organization (WHO) subsequently declared this a pandemic due to its widespread infectivity and high rate of transmission [1]. Early in the pandemic, efficacious vaccines were considered essential for preventing morbidity and mortality caused by COVID-19, and this drove notable advances in the development of new vaccines in a very short time. Less than one year after the SARS-CoV-2 genetic sequence was released by the Chinese Centre for Disease Control and Prevention, Pfizer-BioNtech, Moderna, and AstraZeneca-Oxford University published interim results on the safety and efficacy of their respective vaccines, and were granted emergency use authorisations in the USA, Europe, and elsewhere [2]. At the time, COVID-19 vaccines were first administered, only preliminary reports on clinical trials had been published. These studies assessed the safety, immunogenicity, and efficacy of both mRNA and viral vector vaccines, and concluded that both were safe, well-tolerated, and immunogenic, with efficacies ranging from 70% to 94%, depending on the vaccine type and manufacturer [3,4,5]. Nevertheless, further studies were needed to confirm these preliminary findings due to important limitations of the data.

At the beginning of December 2020, the Portuguese Government released the vaccination plan for COVID-19, which defined priority groups and respective stages of vaccination. In the first phase of the vaccine rollout, priority was given to people older than 50 years of age with long-term clinical conditions, residents in care homes, frontline health and social care workers, and security forces. The second and third phases of vaccination were applied successively to the remaining population according to age and associated pathologies [6]. Two types of vaccines were administered—messenger RNA vaccines and viral vector vaccines. At this point in time, several key questions remained unanswered due to the short duration of follow-up studies [3,4,5]. Knowledge about the duration of SARS-CoV-2 antibodies elicited by vaccination was initially limited to a maximum of 57 days, reflecting the observation period reported in early clinical trial publications. Differences in antibody response induced by vaccines or natural COVID-19 disease were unknown, as were the responses after vaccination of naïve versus Post-COVID recipients. There were also strategic considerations that could impact the success of vaccine programmes, logistical and biological, including vaccine transportation and storage, administration characteristics, and differences in underlying medical conditions of the general population compared to people in clinical trials [7]. Furthermore, as time progressed, it was important that the vaccine covered new variants. The continued emergence of new SARS-CoV-2 variants, particularly the Delta variant (B.1.617.2), caused sharp increases in infections worldwide, including countries with high vaccination coverage [8]. For this reason, the administration of a third dose of COVID-19 vaccine was recommended to address the potential waning of immunity over time and the reduced effectiveness of the vaccine against the Delta variant [9]. The European Medicines Agency (EMA) published its regulatory considerations on heterologous primary and booster COVID-19 vaccination in December 2021 based on evidence generated from short-term immunogenicity studies and a vaccine effectiveness study [10,11].

At the end of December 2020, the Local Health Unit Póvoa de Varzim/Vila do Conde (HPVVC) started immunising health professionals, prioritised according to the risk of exposure. The vaccination schedules applied respected the rules defined by the General Directorate of Health for Portugal, which were as follows: COMIRNATY^®^ vaccine—two doses administered with an interval of 21 to 28 days; VAXZEVRIA^®^ vaccine—two doses administered 8 to 12 weeks apart; and for people who had COVID-19—one dose of mRNA vaccine administered at least 3 months after recovery. To assess the longevity of the antibody response to vaccination, knowing that antibodies against other coronaviruses decrease over time (range: 12–52 weeks from the onset of symptoms) [12], to compare the humoral immune response to the mRNA vaccine versus the viral vector vaccine, and to compare infection versus vaccination, we conducted a prospective cohort study (observational study) enrolling healthcare workers, both naïve and previously infected. We report the quantity and duration of the humoral immune response after vaccination up to two months after the booster dose.

## 2. Materials and Methods

### 2.1. Study Design and Participants

**Figure 1:**
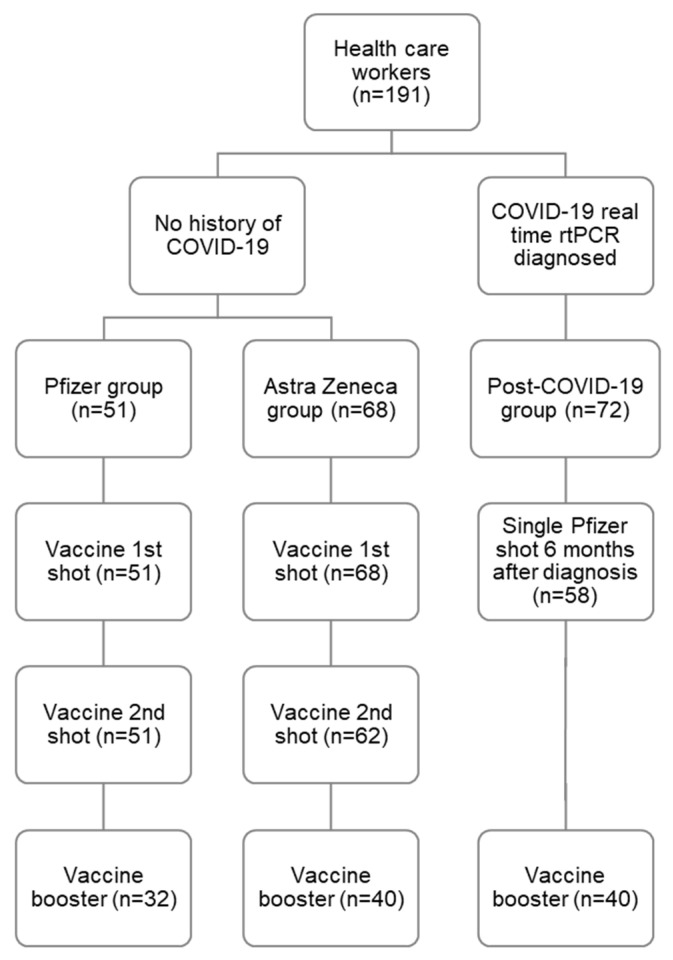
Graphical representation of the prospective cohort study with timepoints of vaccine administration and number of participants at each timepoint.

Anti-SARS-CoV-2-S RBD antibody levels were monitored over time using blood samples collected from 191 hospital workers vaccinated with the Pfizer-BioNTech COVID-19 (COMIRNATY) and the Oxford-AstraZeneca vaccines (Vaxzevria). The period of data collection spanned from January 2021 to March 2022. All participants volunteered and consented to take part in the study. Blood was drawn before administration of the first and second doses, and one month, four months, and eight months (only the Pfizer-BioNTech vaccine group) after full vaccination. At the end of 2021, the booster dose was administered, and the last evaluation took place two months later. Descriptions of the study’s vaccine administration and follow-up are summarised in Figure 1 and Figure S1. All participants completed questionnaires at the time of blood sample collection, which included demographic information and previous history of COVID-19.

### 2.2. Sample Processing and Analysis

Blood samples were collected in Sarstedt S-Monovette tubes, SARSTEDT AG & Co. KG, Nümbrecht, Germany (order number 01.1602.001), and centrifuged at 3000 rpm for 10 min. Sera were transferred to new test tubes and underwent antibody testing. Antibodies directed at the spike protein of SARS-CoV-2 were measured at each timepoint using Roche Elecsys^®^ anti-SARS-CoV-2 S kit, F. Hoffmann-La Roche Ltd, Basel, Switzerland (order number 09289267190), and Cobas e411 analyser F. Hoffmann-La Roche Ltd, Basel, Switzerland [13]. Elecsys^®^ Anti-SARS-CoV-2 S is an electrochemiluminescence immunoassay for the in vitro quantitative determination of antibodies (including IgG) to the SARS-CoV-2 spike (S) protein receptor binding domain (RBD) in human serum and plasma. The assay uses a recombinant protein representing the RBD of the S antigen in a double-antigen sandwich assay format, which favours the detection of high-affinity antibodies against SARS-CoV-2. Samples with anti-SARS-CoV-2-S RBD concentrations above the measuring range (250 U/mL) were diluted automatically by the analyser with Diluent Universal, from 1:10 up to 1:100. After dilution by the analyser, the software automatically takes the dilution into account when calculating the sample concentration. Results were reported as concentrations (U/mL), with a cut-off of 0.8 U/mL or more considered positive.

### 2.3. Statistical Analysis

The serological assay values were summarised using medians, ranges and geometric means. Differences across timepoints and vaccines were assessed using Kruskal–Wallis and Wilcoxon non-parametric tests. Multiple linear regression analysis was further used on log transformed ratios of serological assay values to assess differences across vaccine groups and time adjusting for sex and age. A linear mixed model was applied to (log) antibody levels with fixed effects for age, gender, vaccine, time, and time–vaccine interactions, with a random effect for participant. The R statistical analysis tool (v.4.4.1) was used to perform the analysis (R code may be made available upon request from the corresponding author). The baseline data were complete, and the statistical analyses were unaffected by missing data. Some participants were lost to follow-up, and were not replaced. We report the sample sizes across time, and adopted a complete case analysis approach.

## 3. Results

### 3.1. Participants

Between January 2021 and March 2022, 191 healthcare workers were followed up after the first COVID-19 vaccination dose. The cohort included physicians, nurses, nurse aids, paramedical personnel, administrative, and logistic employees. The mean age at vaccination was 43 years (median: 42; range 20–68 years). Most participants were female (160/191, 83,8%). Participants were split into three groups: Oxford-AstraZeneca (Vaxzevria) vaccine group (AZ: *n* = 68, 35.6%), Pfizer-BioNTech COVID-19 (COMIRNATY) vaccine group (Pfizer: *n* = 51, 26.7%), and Post-COVID group (*n* = 72, 37.7%) (Table 1). The age and sex distributions were similar across the three groups (Chi-squared test *p* > 0.2). The Post-COVID group received one dose of the Pfizer-BioNTech COVID-19 (COMIRNATY) vaccine ~six months after SARS-CoV-2 infection. This group consisted of nurses (22, 30.3%), healthcare assistants (17/72; 23.6%), medical doctors (16, 22.2%), and other health professionals (17, 23,6%). The main symptoms of this group were as follows: muscle pain (40/72), headache (38), loss of taste and smell (34), cough (26), fever (25), nausea (13), sore throat (12), and other (30).

### 3.2. Concentration of Antibodies Against the SARS-CoV-2 Spike RBD over Time

The levels of antibodies were quantified at baseline (day of first dose = T0) and three weeks after the first vaccination for the Pfizer-BioNTech vaccine group (day of second dose = T1); at baseline (day of first dose = T0) and three months after the first vaccination for the AZ vaccine group (day of second dose = T1); at baseline (day of first dose = T0) for the Post-COVID group (only one dose); and during the respective follow-up (Table 1).

At baseline (T0), the median titre of antibodies against SARS-CoV-2 S-RBD was 0.4 UI/mL in both Pfizer and AZ groups; nevertheless, there were participants with evidence of previous asymptomatic infection (N with >0.8 UI/mL: Pfizer 4, AZ 8). The lowest antibody level in the Post-COVID group was 3.65 UI/mL, with all others >10 UI/mL. The median titre in the Post-COVID group was 97.9 UI/mL (IQR 46.79–192.43), which is similar to the median titre observed at T1 (day the second vaccine dose) in the Pfizer (93.1) and AZ (72.6) groups.

**Figure 2:**
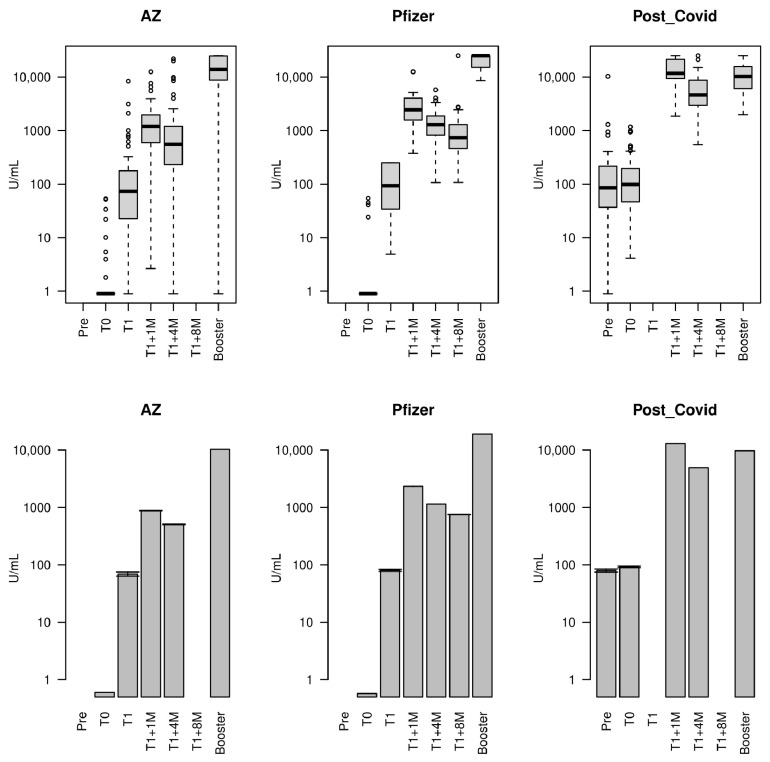
Serology profiles across timepoints. The top row are boxplots of serological data, and bottom row show geometric means (+/− 1 standard deviation); AZ = AstraZeneca; T0 = baseline (first vaccine); T1 = second vaccine shot (except Post-COVID group), which is 3 weeks and 3 months after T0 for the Pfizer (T1a) and AZ (T1b) groups, respectively; T1 + 1M = T1 + 1 month to baseline; T1 + 4M = T1 + 4 months to baseline; T1 + 8M = T1 + 8 months to baseline (only Pfizer group); Booster = third dose. For the Post-COVID group, T1 + 1M~T0 + 1M and T1 + 4M~T0 + 4M.

The trends in the antibody levels against SARS-CoV-2 S-RBD during the follow-up period were similar amongst the groups. Namely, there was a spike in the immune response within one month of full vaccination, followed by a progressive decline, requiring a booster (Table 2; Figure 2).

High titres of antibodies were detected one month after full vaccination (Tone: T1 + 1m) for the three groups, although statistically significant differences exist between them (median (IQR): Post-COVID 11,703 (9406–21,368), AZ 1197 (592–1964), Pfizer 2441 (1564–4030)) (Kruskal–Wallis test *p* < 2.2 × 10^−16^). In particular, those in the Post-COVID group were the highest, 4.8 and 9.8 times higher than the medians of the Pfizer and AZ groups, respectively, whilst the levels in the Pfizer group were 2.0 times higher than those in the AZ group (Wilcoxon test *p*-value < 0.00002). Compared to baseline, the antibody levels at Tone were orders of magnitude greater, with the Post-COVID group having the least change due to already being exposed to the virus (T1 + 1M/T0 ratio median (IQR): AZ 2362 (645–3741); Pfizer 5855 (3268–8353); Post-COVID 129 (60–222)) (Kruskal–Wallis *p* < 2.2 × 10^−16^). Antibody titres declined four months after the second dose (T1 + 4M) for all participants. The median antibody titre of the Pfizer group remained 2.3 times higher than that of the AZ group, whilst the Post-COVID group median titre was 3.6 and 8.4 times higher than that of Pfizer and AZ groups, respectively. Between T1 + 1M and T1 + 4M (i.e., ~3 months difference) there were decreases of ~60% in antibody levels, with Pfizer appearing to decrease the least (median: Pfizer 46%; AZ 60%; Post-COVID 65%) (Kruskal–Wallis *p* = 0.0033) (Table 2; Figure 2).

**Figure 3:**
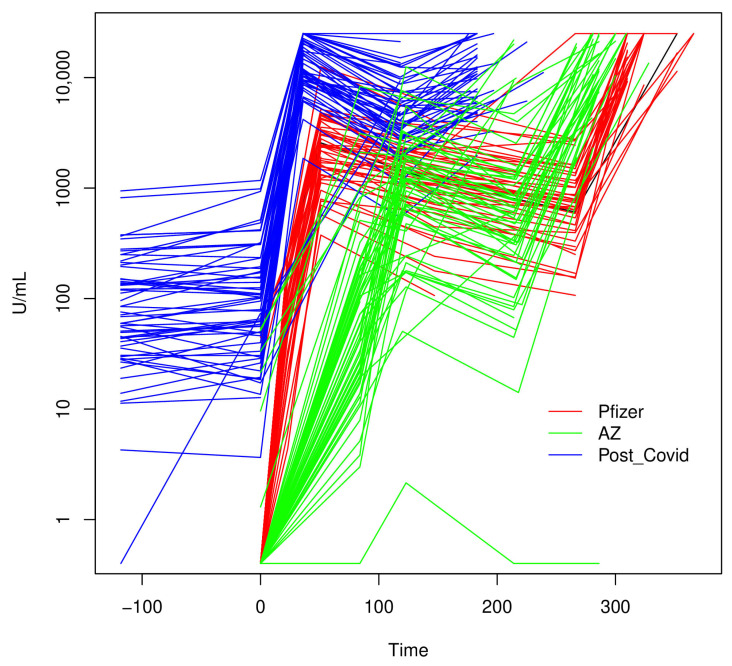
The serological profiles of participants across time, where time zero (T0) is the baseline.

The decline in antibody levels was interrupted by the administration of the third dose. Unlike other timepoints, there was incomplete sampling at the booster timepoint (number with serology: AZ 40/68; Pfizer 32/51; Post-COVID 40/72). On average, 56 days after the booster (median (IQR) days: 56 (49–70)), compared to the T1 + 4M timepoint, the booster median increases in antibody levels were 25-fold for AZ, 19-fold for Pfizer, and 2-fold for Post-COVID groups (Table 2; Figure 2). Whilst both the boosters for the AZ and Pfizer groups led to antibody levels higher than those at any previous sample collection point (median (IQR): AZ 13,846 (9043–24,555), Pfizer 25,000 (15,220–25,000)), the booster for the Post-COVID group led to antibody levels lower than those at the T1 + 1M timepoint (median (IQR): 10,264 (6111–15,552)). Notably, the Pfizer group levels were significantly higher (Wilcoxon *p* <0.0005). One participant did not respond to vaccination (in the AZ group with Pfizer booster). An estimated 12 participants contracted COVID-19 during the follow-up (Pfizer 3, AZ 7, Post-COVID 2), as detected by a sharp rise in antibody titre (Figure 2 and Figure 3).

Using multivariate linear regression models, we considered the four common timepoints across each vaccine group (T0, T1 + 1M, T1 + 4M, Booster), and modelled the (log) ratio of the antibody levels relative to the T1 + 1M (Table 3). Consistently with the results above, the Post-COVID group had higher antibodies at the baseline (T0) ratio, and whilst the Pfizer group had higher nominal effects at T1 + 4M and booster timepoints, the ratios to Tone were similar between AZ and Pfizer groups (*p* > 0.03). Importantly, the Post-COVID group did not respond as well to the booster as the AZ and Pfizer groups (*p* < 0.001), which could point to antibody feedback (Figure 3, Table 3). There were no strong effects of age (*p* > 0.39) or sex (*p* > 0.29) on the ratios.

A linear mixed model was applied to (log) antibody levels with fixed effects for age, gender, vaccine, time (4 common timepoints), and time–vaccine interactions, with a random effect for the participants. Overall, there were significant time, vaccine, and vaccine-time interaction effects (all F-test *p* < 2 × 10^−16^), but not for age (*p* = 0.11) or gender (0.93), all of which were consistent with previous analyses (Table 4).

## 4. Discussion

Our study focused on the kinetics of the humoral immune response to COVID-19 vaccines in three different groups of healthcare workers. Two groups were composed of participants with no previous history of SARS-CoV-2 infection who were vaccinated with either two doses of Pfizer-BioNtech vaccine or two doses of Oxford-AstraZeneca vaccine. A third group was composed of participants previously infected with SARS-CoV-2 and vaccinated with one dose of Pfizer-BioNtech vaccine. All groups received a booster dose of the Pfizer-BioNtech vaccine.

When the first vaccine dose was given (T0), four volunteers in the Pfizer group and eight in the AZ group had antibody levels indicating prior infection with SARS-CoV-2. Since these participants stated in the vaccination survey that they had not previously contracted COVID-19, the infection was probably asymptomatic. During the post-vaccination monitoring period, three participants in the Pfizer group, seven in the AZ group, and two in the Post-COVID group experienced a sharp increase in antibody levels at T1 + 4 months after full vaccination, despite the expected decline in titres by this stage. Due to this, we concluded that these individuals were infected with SARS-CoV-2 post-vaccination, likely without symptoms as they did not disclose their condition during the booster appointment.

Our study was initially impacted by the emergence of the Alpha variant, followed by the Delta variant, and finally by the Omicron variant. The vaccines administered in the first, second, and third doses, designed for the original Wuhan strain of SARS-CoV-2, provided good protection against the disease caused by the variants that emerged up to Delta. Vaccination reduced the positivity duration and viral load in infected patients compared to unvaccinated patients. The illness was also reported to be mild [14]. Vaccine immune evasion and breakthrough infections became common due to the emergence of Omicron. The vaccine effectiveness was lower against this strain. Yet, the influence of the Omicron variant on our research was limited since it emerged towards the end of our observation period.

The kinetics of antibody production observed in our study, namely, high titres reached approximately one month after full vaccination followed by a progressive decline, are corroborated by findings reported in other studies. For example, one study found that total IgG anti-S protein responses assessed at different timepoints for three months in 871 participants vaccinated with two doses of Pfizer-BioNtech continuously increased, peaking after the second dose. Subsequently, a significant decrease occurred in the third month [15]. Similarly, a research conducted over six months found that the IgG produced in response to Pfizer-BioNtech peaked at two months and decreased over the next four months [16]. These observations were corroborated by several other similar investigations [17,18,19]. The Oxford-AstraZeneca vaccine was also assessed in real-world studies, which revealed that following the second dose, IgG anti-S protein levels peaked after 21 days and gradually decreased thereafter. Nevertheless, IgG antibodies could be detected in the serum for up to six months [20,21,22,23,24].

We also observed that peak antibody levels were higher following mRNA vaccination than following viral vector vaccination or natural infection, consistently with previous work [25,26]. In addition, homologous and heterologous vaccination schedules with Pfizer-BioNtech have been found to be more immunogenic than the homologous Oxford-AstraZeneca schedule. Also, no other immunisation plan generated neutralising antibodies greater than those induced by the homologous Pfizer-BioNTech schedule [27]. Another study analysed SARS-CoV-2-specific T cells and antibodies in transplant recipients and controls after homologous or heterologous vaccine regimens. Antibody titres in the control group were higher after mRNA vaccination, whereas the vector vaccine elicited greater numbers of T cells [28]. Additionally, we have shown that the initial antibody levels post-vaccination are much higher than those post-infection. A large-scale retrospective study following Pfizer-BioNtech mRNA vaccine administration also revealed that vaccinated individuals had higher antibody titres after the second dose than did convalescent individuals [29]. This observation confirmed findings from two small-scale studies that had reported initial antibody levels post-vaccination to be much higher than post infection [30,31].

Our results indicate that there are different kinetics of antibody production between individuals who received the Pfizer-BioNtech mRNA vaccine and those who received the Oxford-AstraZeneca vector vaccine, or individuals who had COVID-19 before being vaccinated with one dose of the Pfizer-BioNtech vaccine. The several classes of COVID-19 vaccines are different in their mode(s) of action, and in the way the spike antigen is presented to the immune system [32]. Pfizer-BioNtech and Oxford-AstraZeneca are mRNA and adenoviral vaccines, respectively, both of which provide genetic information for the biosynthesis of the spike protein (S) in body cells of vaccines [33,34]. These vaccines require that properly formed and folded S proteins are presented to B cells for the production of antibodies that successfully neutralise the virus [32]. Moreover, mRNA and adenovirus-vector vaccines stimulate different aspects of the immune response, particularly the innate immune response [35]. Variations in the biosynthesis of the S protein and antigen presentation may lead to differences in immunogenicity, as observed in our study.

Our work has shown that persons without a history of COVID-19 produce significantly lower antibody titres after the first and second doses of vaccination than individuals who were vaccinated after infection. Additionally, we observed that the booster administered ~10 months after the second dose led to an increase in antibody titres greater than the peak following initial vaccination in the Pfizer and AstraZeneca groups (immune-naïve recipients). Findings from COV-BOOST, a randomised controlled trial that enrolled participants with no history of laboratory-confirmed SARS-CoV-2 infection, demonstrated that vaccines given as the third dose induced significantly higher anti-spike IgG levels at 28 days post boost than did the corresponding controls [36]. Similarly, another study showed that a third dose of Pfizer-BioNtech administered seven to eight months after the second dose resulted in an increase in neutralising antibodies greater than the peak following the primary two-dose series [37]. However, in our cohort, anti-spike antibody levels at approximately 2 months after the administration of the third dose did not increase as expected in individuals with a history of COVID-19 prior to vaccination (median increase from 4610 to 10,246 U/mL, the latter being lower than the median at T1 + 1m), although they increased substantially more in subjects without a history of COVID-19 (median increase from 550.8 to 13,846 U/mL in AZ group and median increase from 737.3 to 25,000 U/mL in Pfizer group). A recent study also revealed a much smaller increase in antibody levels after the third dose in persons with a history of COVID-19, although they continued to have significantly higher levels of anti-SARS-CoV-2 S antibodies than did those without a history of previous infection [38]. We hypothesise that the poor response after the booster in the Post-COVID group may be due to negative feedback from epitope masking, and/or antigen clearance at the site of vaccine injection caused by high levels of pre-existing antibodies. Overall, these findings indicate the modulation of the immune response to COVID-19 vaccines by previously elicited antibodies.

The immune response to an antigen results from various factors, among which the immunological memory of past exposures is included. It has been observed that antibody responses to malaria vaccines can be poor after repeated doses [39,40]. The humoral immunity against the radiation-attenuated *P. falciparum* whole-sporozoite (PfSPZ) vaccine candidate in malaria-naïve humans, studied by profiling B cell and antibody responses, showed that vaccine-specific antibodies increased and peaked after the first and second doses, but plateaued afterwards [41]. In addition, the expansion of specific B cells was lower after the third dose than after the second dose. Notably, subjects with high antibody titres at the time of the booster had poorer responses. These observations suggest that vaccine-induced antibodies bind epitopes masking them from B cell recognition, thus leading to the suppression of memory B cell responses [41].

Epitope masking has also been reported with seasonal and epidemic influenza vaccines. A mathematical model approach to investigate how pre-existing immunity affects the dynamics of antibody responses to influenza revealed that the masking of epitopes by antibody binding may play an important role in the dynamics of recall responses [42,43]. In the presence of epitope masking, there is a decrease in the primary response and a much greater reduction in the boost following subsequent immunisation. This is because the masking effect is greater and the boost is smaller as the amount of antibody prior to immunisation increases. Conversely, in the absence of epitope masking, secondary immunisation results in an increase in antibody titres similar to that observed during the primary response, which is maintained for subsequent immunisations. These mathematical models were tested using published data from vaccination of humans with the 2012/2013 trivalent influenza vaccine [44]. Predictions of how pre-existing antibodies to influenza A hemagglutinin decrease the magnitude of the booster antibody response to this epitope following immunisation were confirmed.

Research indicating that prior immunity affects the immune response to COVID-19 vaccines emerged in late 2022, and early 2023. One study compared the antibody responses of Pfizer-BioNTech vaccine recipients following first, second, and third vaccine doses to the antibody responses of COVID-19 patients [45]. A significant feature was found that distinguishes the antibodies generated by vaccines from those generated after natural SARS-CoV-2 infection: vaccine-induced antibodies have ‘breadth’ and can bind different viral variant spike proteins, whereas natural infection does not generate comparable breadth. Another important finding was the detection of the spike antigen, in 96% of vaccinees, in plasma collected 1–2 days after the prime injection, with levels reaching as high as 174 pg/mL. However, the detection of the spike antigen in plasma samples ceased after the second dose of Pfizer-BioNtech vaccination, likely due to the formation of circulating immune complexes of anti-spike antibodies and spike protein. These complexes would mask antigen epitopes, preventing capture by detection antibodies, which is the basis of antigen detection assays. Similar assay interference has been reported for other diseases [46,47]. Another study using preclinical SARS-CoV-2 mouse models demonstrated that previously elicited antibodies at low titres, or from broad multi-epitope responses, enhanced naïve B cell recruitment to germinal centres. In contrast, high titres of high-affinity mono-epitope-specific antibodies limited the participation of cognate naïve B cells. The directionality and intensity of this effect were determined by the antibody concentration, affinity, and epitope specificity of the primary response [48]. To study the influence of pre-existing antibodies in the development of memory B cells, researchers examined the memory B cell response in healthy individuals who received two high-affinity anti-SARS-CoV-2 monoclonal antibodies and, afterwards, two doses of an mRNA vaccine [49]. The results showed that pre-existing high-affinity antibodies can modify the antibody target profile, affinity, and isotype of the responding cells through two distinct mechanisms, one of which is direct masking of their cognate epitopes. Finally, a study of the anti-SARS-CoV-2 spike protein antibody responses in unexposed and exposed individuals revealed that lower antibody levels after the first dose were associated with a greater increase in antibody levels after the second dose, suggesting that pre-existing antibodies modulate the immunogenicity of mRNA vaccines [50]. In addition, mouse experiments have shown that pre-existing antibodies increase the clearance of vaccine antigens at the site of immunisation, limiting the amount of antigen available to prime B cell responses after mRNA boosters.

Reports of better antibody responses when the initial Pfizer-BioNTech doses are spaced at 12 weeks rather than at 3 weeks [51], and when people who are given initial Oxford-AstraZeneca doses spaced at 3–4 weeks apparently have lower protection against infection than people who received the latter 16 weeks apart [52], are in agreement with the research mentioned above and may also be indicative of epitope masking leading to feedback inhibition.

To assess vaccine effectiveness in real-world settings is paramount. A multicentre study conducted in Europe during the autumn of 2023 estimated the COVID-19 vaccine effectiveness to be 40% overall against laboratory-confirmed symptomatic infections [53]. Another recent study reported a vaccine effectiveness of 60% against hospitalisation of persons with a positive test for SARS-CoV-2 who had received the last dose in the preceding three months. Vaccine effectiveness for severe disease was halved when the last vaccine dose had been administered in the preceding six months. As expected, vaccine effectiveness waned over time and was almost absent after six months [54].

The global SARS-CoV-2 test positivity reported to FluNet in October 2024 was 10.4% across 34 countries of the European Region. Over 312,000 new cases and 803 deaths were reported during the four-week period of the epidemiological update [55].

There are a few limitations of our study. First, the assay we used does not specifically measure neutralising antibodies. However, a high correlation was observed between a surrogate virus neutralisation assay and other assays, such as the Roche Elecsys^®^Anti-SARS-CoV-2 S assay [56,57]. Second, an additional limitation is that the assay uses a recombinant RBD protein for the in vitro quantitative determination of total antibodies in human serum and plasma, which favours the detection of high-affinity antibodies against SARS-CoV-2. Therefore, the Elecsys^®^Anti-SARS-CoV-2 S does not measure antibodies of greater breath directed at other epitopes of the spike protein, which may be produced if epitope masking by excess antibody occurs. Third, the age of the enrolled subjects ranges from 20 to 68 years, which corresponds to the working age of our study population composed of healthcare workers. Therefore, our work does not provide insights into immunity in children.

## 5. Conclusions

Our study showed that the highest antibody titres were reached approximately one month after full vaccination, and steadily declined thereafter. We also observed that mRNA vaccination induces higher titres of antibodies than viral vector vaccination, and both generate greater antibody responses than mild or moderate COVID-19. These observations are consistent with prior reports and point to differences in the immune responses induced by the Pfizer-BioNTech and Oxford-AstraZeneca vaccines as well as the disease. Additionally, we observed that whether initial exposure took place through natural infection or vaccines modulates the response to subsequent vaccination, particularly after the third dose. Our findings point to unwanted feedback where high titres of antibodies hinder vaccine boosting, possibly via epitope masking, as observed after repeated administration of the PfSPZ malaria vaccine.

As we are in the fourth year after the pandemic was declared, most of the world population has had several rounds of vaccination as well as the disease. The outcome of this study has implications for repeated rollout strategies even if they are adapted to new SARS-CoV-2 variants. This study has the potential to inform policy makers and national immunisation committees that need to establish vaccination criteria based on immunological considerations.

## Figures and Tables

**Table 1:** Baseline characteristics.

Characteristic	AZ (*n* = 68) Median (N)	AZ (*n* = 68) Range (%)	Pfizer (*n* = 51) Median (N)	Pfizer (*n* = 51) % (Range)	Post-COVID (*n* = 72) Median (N)	Post-COVID (*n* = 72) % (Range)	Difference *p*-Value ****
Age (years)	44.5	20–66	41	24–65	44	20–68	0.968
Female	60	88.2%	39	76.5%	61	84.7%	0.218
Serology time diff. (days)							
Pre-baseline * to baseline (T0)	··	··	··	··	−118	−118–−118	··
T1a (3 weeks) to baseline	··	··	23	23–23	··	··	··
T1b (3 months) to baseline	84	84–84	··	··	··	··	··
T1 + 1 month to baseline	123	120–123	51	51–51	36	36–36	<0.001
T1 + 4 months to baseline	214	214–221	147	147–147	118	118–118	<0.001
T1a + 8 months to baseline **	··	··	266	266–267	··	··	··
Booster *** to baseline	286	267–328	310	303–366	183	176–239	<0.001

AZ = AstraZeneca; T0 = first vaccine shot (baseline); T1 = second vaccine shot (except Post-COVID group); * blood collected 4 months before vaccination (only Post-COVID group); ** 8 months (only Pfizer group); *** there was incomplete sampling at the booster timepoint (number with serology: AZ 40/68; Pfizer 32/51; Post-COVID 40/72); note that T1 + 1 (4) months can be considered ~T0 + 1 (4) months for the Post-COVID group; **** all Kruskal–Wallis tests, except for gender (Chi-Sq).

**Table 2:** Serological profiles across timepoints.

Vaccine	Timepoint	Median	IQR	Geom. Mean (SD)	Fold Change in Geom. Mean [Trend]
AZ	T0	0.400	0.400–0.400	0.60 (3.28)	Baseline [-]
	T1b *	72.57	22.08–173.65	69.33 (5.59)	116.23 [>]
	T1b + 1 month	1197	592.35–1963.6	878.80 (4.16)	12.68 [>]
	T1b + 4 months	550.8	234.7–1200.2	507.28 (6.05)	0.58 [<]
	Booster	13,846	9042.5–24,554.5	10,251.21 (5.87)	20.21 [>]
Pfizer	T0	0.400	0.400–0.400	0.57 (3.48)	Baseline [-]
	T1a *	93.09	36.84–250	80.17 (3.05)	139.95 [>]
	T1a + 1 month	2441.0	1564–4030	2332.60 (2.04)	29.10 [>]
	T1a + 4 months	1294.0	824.4–1874	1145.33 (2.24)	0.49 [<]
	T1a + 8 months	737.3	463.5–1265.2	754.91 (2.63)	0.66 [<]
	Booster	25,000	15,220–25,000	18,923.95 (1.45)	25.07 [>]
Post-COVID	4 months pre-T0	84.95	36.77–213.85	79.45 (4.70)	-
	T0	97.86	46.79–192.43	92.01 (3.30)	Baseline [-]
	T1 ** + 1 month	11703	9406–21,368	12,977.29 (1.73)	141.04 [>]
	T1 ** + 4 months	4610	3008.8–8450	4920.43 (2.09)	0.38 [<]
	Booster	10,264	6111–15,552	9702.92 (1.90)	1.97 [>]

IQR = inter-quartile range; Geom. = geometric; SD standard deviation; AZ AstraZeneca (n = 68), Pfizer (n = 51), Post-COVID (n = 72); T0 = baseline (first vaccine shot); * T1 = second vaccine shot (except Post-COVID group), which is 3 weeks and 3 months after T0 for Pfizer (T1a) and AZ (T1b) groups, respectively; ** note that T1 + 1 (4) month(s) can be considered ~T0 + 1 (4) month(s) for the Post-COVID group; there was incomplete sampling at the booster timepoint (number with serology: AZ 40/68; Pfizer 32/51; Post-COVID 40/72).

**Table 3:** Serology measures compared to the T1 + 1-month (T1 + 1M) timepoint.

Timepoint Ratio (/T1 + 1M)	Vaccine	Median	IQR	Mean (SD)	Difference *p*-Value *
T0	AZ	0.0004	0.0002–0.0016	0.0057 (0.0263)	<10^−15^
T0	Pfizer	0.0002	0.0001–0.0003	0.0005 (0.0010)	
T0	Post-COVID	0.0078	0.0045–0.0166	0.0115 (0.0106)	
T1 + 4M	AZ	0.4033	0.3185–0.5172	0.4170 (0.1623)	0.0033
T1 + 4M	Pfizer	0.5402	0.3517–0.6536	0.5283 (0.1971)	
T1 + 4M	Post-COVID	0.3645	0.2903–0.4905	0.4045 (0.1770)	
Booster	AZ	12.924	7.812–27.356	22.165 (27.088)	<10^−15^
Booster	Pfizer	7.8852	5.942–10.484	9.759 (6.612)	
Booster	Post-COVID	0.8558	0.5752–1.000	0.8787 (0.4360)	

IQR = inter-quartile range; AZ = AstraZeneca; * Kruskal—Wallis test; T0 = Baseline (first vaccine shot); T1 = second vaccine shot (except Post-COVID group), which is 3 weeks and 3 months after T0 for the Pfizer (T1a) and AZ (T1b) groups, respectively (see Table 1 and Table 2); T1 + 1M = T1 + 1 month to baseline; T1 + 4M = T1 + 4 months to baseline; Booster = third dose. For the Post-COVID group, T1 + 1M~T0 + 1M and T1 + 4M~T0 + 4M; there was incomplete sampling at the booster timepoint (number with serology: AZ 40/68; Pfizer 32/51; Post-COVID 40/72).

**Table 4:** Linear mixed model of (log) serological assay values across timepoints.

Fixed Effects	Estimate	LCL	UCL	*p*-Value
Intercept	0.6716	0.1743	1.1689	0.0088
Age	−0.0083	−0.0183	0.0018	0.1103
Male vs. female	0.0152	−0.3083	0.3386	0.9270
T1 + 1M vs. T0	6.5991	6.3190	6.8793	<2 × 10^−16^
T1 + 4M vs. T0	6.0483	5.7734	6.3232	<2 × 10^−16^
Booster vs. T0	9.2521	8.9481	9.5560	<2 × 10^−16^
Pfizer vs. AZ	−0.0336	−0.4009	0.3337	0.8578
Post-COVID vs. AZ	4.1967	3.8421	4.5512	<2 × 10^−16^
T1 + 1M × Pfizer *	0.8963	0.4972	1.2955	0.00001
T1 + 4M × Pfizer *	0.7621	0.3607	1.1635	0.00023
Booster × Pfizer *	0.3556	−0.0918	0.8031	0.1200
T1 + 1M × Post-COVID *	−1.6839	−2.0763	−1.2916	<7 × 10^−16^
T1 + 4M × Post-COVID *	−2.0898	−2.4749	−1.7047	<2 × 10^−16^
Booster × Post-COVID *	−4.6049	−5.0305	−4.1793	<2 × 10^−16^

AZ = AstraZeneca; T0 baseline; T1 + 1M = T1 + 1 month to baseline; T1 + 4M = T1 + 4 months to baseline; Booster = third dose. For the Post-COVID group, T1 + 1M~T0 + 1M and T1 + 4M~T0 + 4M. LCL, lower 95% confidence interval, UCL, upper 95% confidence interval; * interaction effects.

## Data Availability

The study design is provided in the Supplementary Materials. Due to privacy restrictions, the datasets analysed during the current study will be made available from the corresponding author upon request, and after anonymization.

## Supplementary Materials

The following supporting information can be downloaded at: www.mdpi.com/article/10.3390/vaccines12121358/s1. Figure S1. (A) Follow-up timeline after COVID-19 vaccination. T0 is baseline (first vaccination shot) and T1 is second vaccination shot; Pfizer and AZ groups. (B) Follow-up timeline after COVID-19 vaccination. T0 is baseline (first vaccination shot). Post-COVID-19 group. (C) SARS-CoV-2 variants across the study.
