# Supplementary material for: Longitudinal Immunological Analysis of Portuguese Healthcare Workers Across the COVID-19 Pandemic Reveals Differences in the Humoral Immune Response to Vaccines
Source: Vaccines (Basel). 2024 Nov 30;12(12):1358. doi: 10.3390/vaccines12121358 (PMC11680130; doi:10.3390/vaccines12121358)

(A) Follow-up timeline after COVID-19 vaccination. T0 is baseline (first vaccination shot) and T1 is second vaccination shot; Pfizer and AZ groups

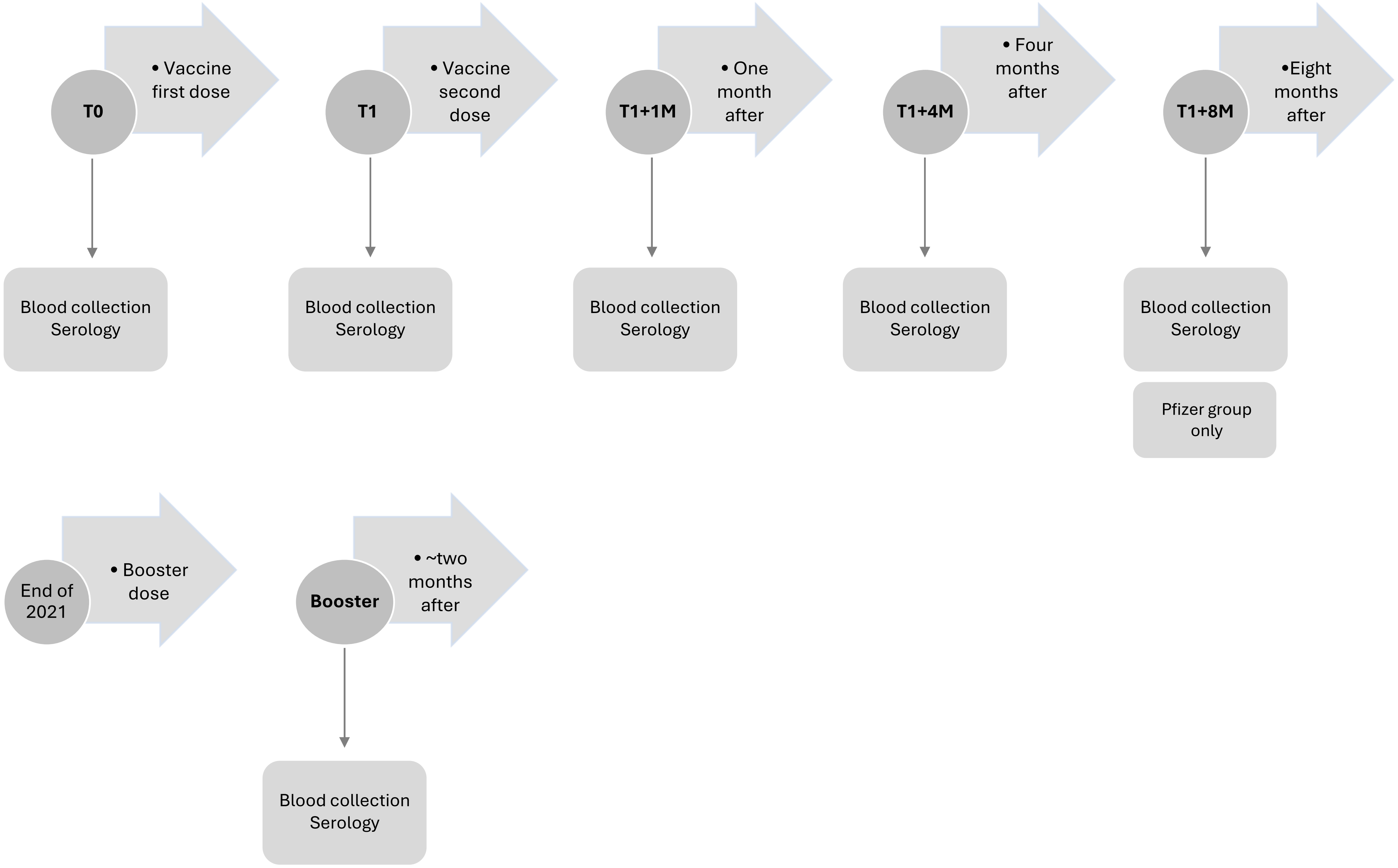

(B) Follow-up timeline after COVID-19 vaccination. T0 is baseline (first vaccination shot). Post-COVID-19 group

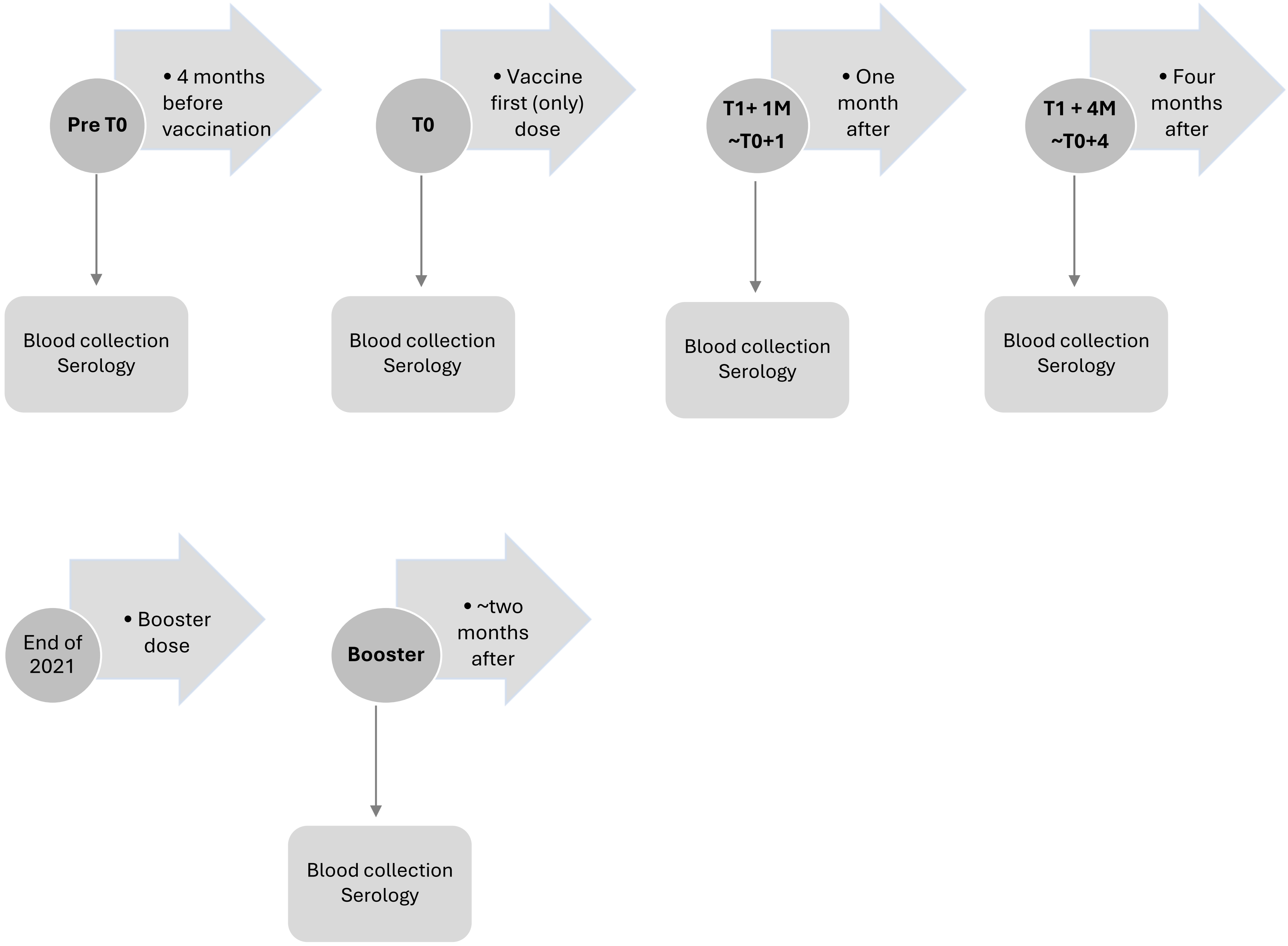

(C) SARS-CoV-2 variants across the study

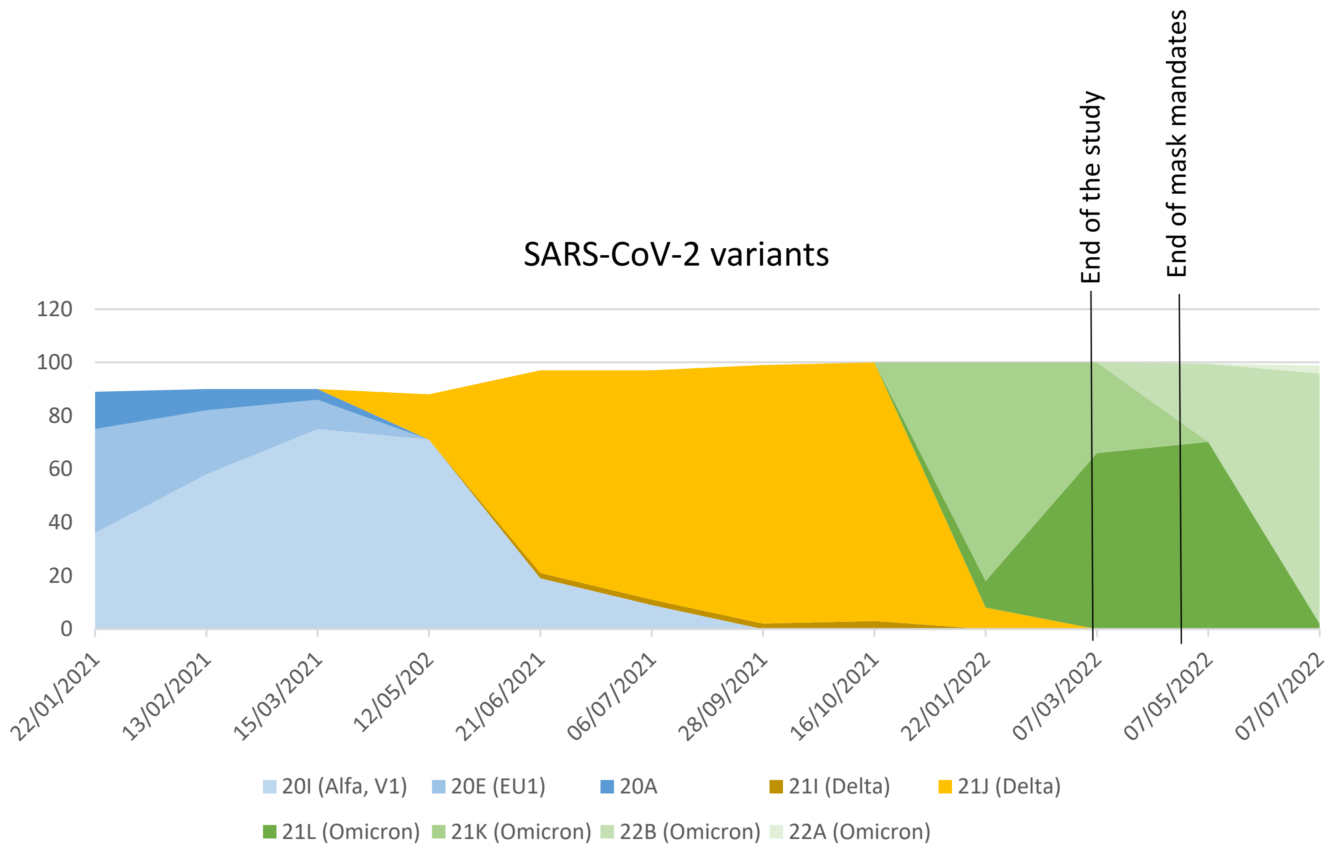

Supplement: Supplementary file 1 [file vaccines-12-01358-s001.zip › vaccines-3249284-supplementary.pdf]
